# Efficacy and safety of metformin for melasma treatment: a systematic review and meta-analysis

**DOI:** 10.3389/fphar.2023.1281050

**Published:** 2023-12-13

**Authors:** Pajaree Mongkhon, Chidchanok Ruengorn, Ratanaporn Awiphan, Chabaphai Phosuya, Yongyuth Ruanta, Kednapa Thavorn, Sirinda Jamjanya, Mati Chuamanochan, Surapon Nochaiwong

**Affiliations:** ^1^ Pharmacoepidemiology, Social and Administrative Pharmacy (PSAP) Research Unit, School of Pharmaceutical Sciences, University of Phayao, Phayao, Thailand; ^2^ Pharmacoepidemiology and Statistics Research Center (PESRC), Faculty of Pharmacy, Chiang Mai University, Chiang Mai, Thailand; ^3^ Department of Pharmaceutical Care, Faculty of Pharmacy, Chiang Mai University, Chiang Mai, Thailand; ^4^ Ottawa Hospital, Ottawa Hospital Research Institute, Ottawa, ON, Canada; ^5^ Institute of Clinical and Evaluative Sciences, ICES uOttawa, Ottawa, ON, Canada; ^6^ School of Epidemiology and Public Health, Faculty of Medicine, University of Ottawa, Ottawa, ON, Canada; ^7^ Institute of Dermatology, Bangkok, Thailand; ^8^ Division of Dermatology, Department of Internal Medicine, Faculty of Medicine, Chiang Mai University, Chiang Mai, Thailand

**Keywords:** melasma, metformin, pigmentation, meta-analysis, triple combined cream

## Abstract

**Objective:** Metformin has recently been demonstrated to have an anti-melanogenic activity. Nevertheless, clinical evidence of the effectiveness of metformin in melasma is lacking. The objective of this study was to assess the efficacy and safety of metformin in the treatment of melasma.

**Methods:** MEDLINE, Embase, PubMed, Cochrane Library (CENTRAL), Scopus, CINAHL, and grey literature databases were searched to 4 October 2022 and updated on 26 February 2023. Randomized controlled trials (RCTs), quasi-RCTs, observational studies, case series, and case reports investigating the efficacy and safety of metformin for melasma were included. The Melasma Area Severity Index (MASI) scores that changed from baseline were pooled using fixed-effects model and expressed as standardized mean differences (SMDs) and 95% confidence intervals (CIs).

**Results:** Three RCTs including 140 patients with melasma were included. The results demonstrated that after 8 weeks, 15% topical metformin significantly reduced the Melasma Area Severity Index (MASI) score compared to placebo (1 trial; *n* = 60; MD, −0.56; 95% CI, −1.07 to −0.04; *p* = 0.034). Furthermore, when compared to triple combination cream (TCC), 30% topical metformin demonstrated similar efficacy in reducing the MASI score after 8 weeks (2 trials; *n* = 80; MD, 0.19, 95% CI, −0.25 to 0.63; *p* = 0.390). Patients using 30% topical metformin had fewer adverse events compared to TCC users, although no statistical difference was found.

**Conclusion:** Topical metformin was as effective as triple combination cream (TCC) in decreasing changes in the MASI score in patients with melasma, with minimum adverse events. Further studies with larger sample sizes, longer follow-up times, and well-designed trials are required.

**Systematic Review Registration:** Identifier PROSPERO (CRD42022351966).

## 1 Introduction

Melasma is an acquired pigmentary condition that commonly affects the face. This condition is common in women and is primarily caused by ultraviolet exposure, sex hormones, and skin inflammation (Sanchez et al., 1981; Espósito et al., 2022; Wang et al., 2022). Melasma is a clinical diagnosis based on symmetric reticulated hypermelanosis in three distinct facial patterns, centrofacial, malar, and mandibular, resulting from increased melanocyte activity and melanin deposition in the skin (Ogbechie-Godec and Elbuluk, 2017). Melasma prevalence ranges from approximately 1% in the general population to 9%–50% in high-risk populations (Taylor, 2003; Moin et al., 2006; Rathore et al., 2011; Ogbechie-Godec and Elbuluk, 2017). Although melasma is asymptomatic, it is a disfiguring disease that has a negative impact on the quality of life and self-esteem of those suffering from it (Jiang et al., 2017). It is also challenging to treat, has a high recurrence rate, necessitating therapy, and should not be overlooked.

Treatment options for melasma target various aspects of melasma pathogenesis, including photodamage, inflammation, vascularity, and pigmentation (Ogbechie-Godec and Elbuluk, 2017). Topical treatments are the first-line therapies for melasma, and concomitant use of such treatments with different mechanisms is preferred over monotherapy. Currently, triple combination cream (TCC) or Kligman’s formula with hydroquinone, tretinoin, and a topical steroid is considered the gold standard topical treatment for melasma owing to its potent and rapid whitening effect (Ferreira Cestari et al., 2007; Cestari et al., 2009; Cassiano et al., 2022). A previous review revealed that TCC is more effective than hydroquinone alone or in combination (Rajaratnam et al., 2010). However, TCC is not recommended for pregnant or breastfeeding women. Indeed, long-term TCC use also causes adverse effects such as allergic and irritative contact dermatitis, redness, burning, and telangiectasias (Cassiano et al., 2022). Effective treatment with fewer unfavorable side effects is needed in long-term melasma therapy.

Metformin is an oral anti-hyperglycemic medication that has been used to treat type 2 diabetes. It also showed lipid-lowering and platelet anti-aggregating effects, indicating that it has a diverse set of pharmacological properties (Badr et al., 2013). Furthermore, metformin plays an important role in cutaneous disorders such as allergic contact dermatitis, hidradenitis suppurativa, and acanthosis nigricans (Chang and Choi, 2020). *In vitro* and *in-vivo* studies have recently demonstrated the anti-melanogenic activity of topical metformin. It has been shown to decrease melanin content in melanoma cells and normal human melanocytes via a cyclic adenosine monophosphate (cAMP)-dependent pathway, which correlates with the decreased expression of melanogenesis master genes. These data suggest that metformin may have a clinical benefit in the treatment of hyperpigmentation disorders (Belisle and Park, 2014; Lehraiki et al., 2014). However, there is no conclusive evidence of the effect of metformin on melasma. Therefore, this study aimed to evaluate the efficacy and safety of metformin compared to those of other treatment options in patients with melasma.

## 2 Material and methods

This systematic review followed the Preferred Reporting Items for Systematic Reviews and Meta-analyses (PRISMA) statement (Supplementary Appendix S1) (Page et al., 2021). The pre-specified protocol was registered in PROSPERO (CRD42022351966).

### 2.1 Data source and study selection criteria

MEDLINE, Embase, PubMed, Cochrane Library (CENTRAL), Scopus, and CINAHL, were searched without language restrictions from inception to 4 October 2022 and updated on 26 February 2023. We supplemented the search with grey literature from Google Scholar, the clinical trial register, OpenGrey, and preprint reports. The reference lists of the included studies, previous systemic reviews, and conference abstracts from dermatology scientific meetings were reviewed to identify relevant studies. The search strategy was based on a combination of keywords, Emtree terms, and Medical Subject Headings terms related to metformin and melasma (Supplementary Tables S1, S2).

References were imported into the reference manager (EndNoteTM 20.1). Two investigators (PM and SN) independently screened titles and abstracts. Subsequently, potentially relevant full-text articles were evaluated according to the eligibility criteria. Disagreements among investigators were resolved through discussion.

Randomized controlled trials (RCTs), quasi-RCTs, observational studies, case series, and case reports were included if they investigated the efficacy and safety of metformin compared to those of placebo or an active comparator, different dosage regimens, or usual care in patients with melasma aged 18 years or more.

Metformin, administered via any route was considered in the present study. We excluded studies that (i) recruited patients with post-inflammatory hyperpigmentation, neurodermatitis, eczema, atrophy, rosacea, or pregnant/lactating women; (ii) had no control group; (iii) had a ≤1 week follow-up period; and (iv) were *in-vitro*/*in-vivo*, animal studies, or reviews. A summary of the inclusion/exclusion criteria is described in Supplementary Table S3.

### 2.2 Outcomes, data extraction, and quality assessment

The efficacy outcome was the change in melasma severity from baseline as measured by the Melasma Area and Severity Index (MASI) score or global severity score. Additional efficacy outcomes included improvement and treatment satisfaction. The improvement was assessed by the improvement percentage score and graded according to the global improvement scale: grade 1, ≤25% improvement (mild); grade 2, 25%–50% improvement (moderate); grade 3, 51%–75% improvement (marked); and grade 4, >75% improvement (near-total/total). Treatment satisfaction was achieved by patients based on their level of satisfaction, which was scored on a 4-point scale as 0 = no improvement, 1 = poor, 2 = slightly satisfied, 3 = satisfied, and 4 = highly satisfied. Any adverse events, tolerability (drop out from adverse events), and unacceptability of treatment (drop out from any causes) were considered safety outcomes. Additional outcomes included patient-reported quality of life and psychosocial aspects, such as depressive symptoms, anxiety, distress, and wellbeing. Two reviewers (PM and SN) extracted the pre-specified data independently using a standardized approach to gather information as follows: (i) study characteristics; (ii) patient characteristics; (iii) intervention and control; and (iv) outcomes of interest (Table 1).

**TABLE 1:** Baseline characteristics from included trials with metformin for melasma.

Characteristics	No. (%)^a^		
	Ali Mapar and Namdari, (2019): *n* = 60	Banavase Channakeshavaiah and Andanooru Chandrappa (2020): *n* = 40	AboAlsoud et al (2022): *n* = 40
Participant characteristics			
Mean age in year ± SD; range (min–max)	35.2 ± 7.1; not reported	37.3 ± 8.3; 23–84	>18 (not specified)
Female	60 (100.0)	33 (82.5)	Not reported
Mean duration of melasma in year ± SD; range (min–max)	Not reported	2.7 ± 2.7; 0.1–9.0	2.3 ± 1.6; 0.5–6.0
Severity of melasma: mean MASI score ± SD; range (min–max)	11.2 ± 4.0; 4.8–24.6	7.1 ± 5.4; not reported	14.2 ± 9.0; 1.2–31.7
Family history of melasma	Not reported	17 (42.5)	8 (20.0)
Site of melasma			
Malar	Not reported	Not reported	39 (97.5)
Forehead	Not reported	Not reported	10 (25.0)
Moustache	Not reported	Not reported	7 (17.5)
Trial characteristics			
Country of enrollment	Iran	India	Egypt
Study setting and design	Monocentric, parallel-group	Monocentric, parallel-group	Monocentric, parallel-group
Trial registry	Not reported	CTRI/2018/12/016588	Not reported
Randomization method	Simple random sampling	Simple random sampling	Simple random sampling
Trial blinding	Double-blind (not specified)	Open-label	Open-label
Inclusion criteria	• Female participants who were not on topical treatment for melasma in the last 3 months	• Adult participants who were not on any medications for melasma for at least 2 weeks for topical therapy, 1 month for systemic steroids, or 3 months for cosmetic procedures (i.e., laser ablation, dermabrasion, or peels)	• Adult participants who were not on any medications for melasma for at least 1 month for topical or systemic treatments, or 3 months for cosmetic procedures (i.e., laser ablation, dermabrasion, or peels)
Exclusion criteria	• Male gender	• Pregnant and lactating women	• Pregnant and lactating women
	• Pregnant and lactating women	• Receiving oral contraceptive pills or phenytoin	• Receiving oral contraceptive pills
	• Receiving oral contraceptive pills or photosensitivity drugs (tetracycline, spironolactone, phenytoin, and carbamazepine)	• Had history of renal dysfunction, acne vulgaris, or rosacea	• Had history of renal or liver dysfunction, active acne vulgaris, or rosacea
	• Had history of renal dysfunction (glomerular filtration rate less than 30 mL/min/1.73 m^2^)	• Allergic to the medications trial	• Allergic to the medications trial
	• Atrophy and telangiectasia in the site of melasma		
	• History of drug allergy		
Treatment group	Metformin 15% cream (aqueous phase of metformin powder 15% and oil phase) apply twice daily	Metformin 30% lotion (mixing 30 g of metformin powder [Systopic laboratories pvt.ltd] with 70% alcohol and propylene glycol in 30% weight: volume ratio) apply at night time daily	Metformin 30% cream (crushing metformin 500 mg tablets [Mina Pharm, ARE] with 70% alcohol and propylene glycol in 30% weight: volume ratio) apply at night time daily
Comparison group	Placebo-controlled (not specified)	Active-controlled: triple combination cream (Kligman’s formula; hydroquinone 2% + tretinoin 0.025% + fluocinolone acetonide 0.01%) apply at night time	Active-controlled: triple combination cream (Kligman’s formula; hydroquinone 2% + tretinoin 0.025% + fluocinolone acetonide 0.01%) apply at night time
Co-intervention	Sunscreen of SPF 50 (not specified)	Sunscreen of SPF 30 in the morning time	Sunscreen of SPF 50+ in the morning time
Duration of treatment follow-up	12 weeks	8 weeks	8 weeks
Funding	Research Deputy of Ahvaz Jundishapur University of Medical Sciences, Ahvaz, Iran	Not reported	Not reported
Overall risk of bias	High	High	High

^a^ Values express as number (%) unless otherwise specified.

Abbreviations: MASI, melasma area and severity index; SD, standard deviation; SPF, sun protection factor.

Two reviewers (PM and SN) independently appraised the methodological quality of the included studies using the Cochrane revised tool for assessing the risk of bias (RoB Version 2.0) for RCTs (Sterne et al., 2019). For non-RCT studies, we used the Newcastle-Ottawa Scale (Wells et al., 2023). Studies with a total score of ≥8 were defined as of high quality. Two reviewers (PM and SN) critically appraised the strength of evidence for each outcome using the Grading of Recommended Assessment, Development, and Evaluation (GRADE) guidelines (Balshem et al., 2011). Disagreements were resolved through discussion.

### 2.3 Statistical analysis

For continuous outcomes, when at least two studies were available, mean differences or mean changes from baseline were pooled and expressed as mean differences (MDs). We used the odds ratios (ORs) with 95% confidence intervals (CIs) as the common effect estimates for binary outcomes. We pooled study estimates using the inverse variance method for a fixed-effects model if there was no significant heterogeneity among the studies. However, a random-effects model was used if substantial clinical or statistical heterogeneity was observed (DerSimonian and Laird, 1986). Statistical heterogeneity was assessed using Cochran’s Q test, with *p* < 0.10 (Higgins et al., 2003). The degree of heterogeneity was estimated using I^2^ and tau-squared (τ^2^) statistics, in which the heterogeneity was investigated as low (I^2^<25.0%, (τ^2^ = 0.01), moderate (I^2^ = 25–75%, τ^2^ = 0.06), and high (I^2^>75.0%, τ^2^ = 0.16) (Higgins et al., 2003; Borenstein et al., 2017). Statistical tests were two-sided, with a *p* < 0.05. All analyses were conducted using the STATA software (version 16.0; StataCorp, Stata Statistical Software. College Station, TX: United States).

## 3 Results

**FIGURE 1:**
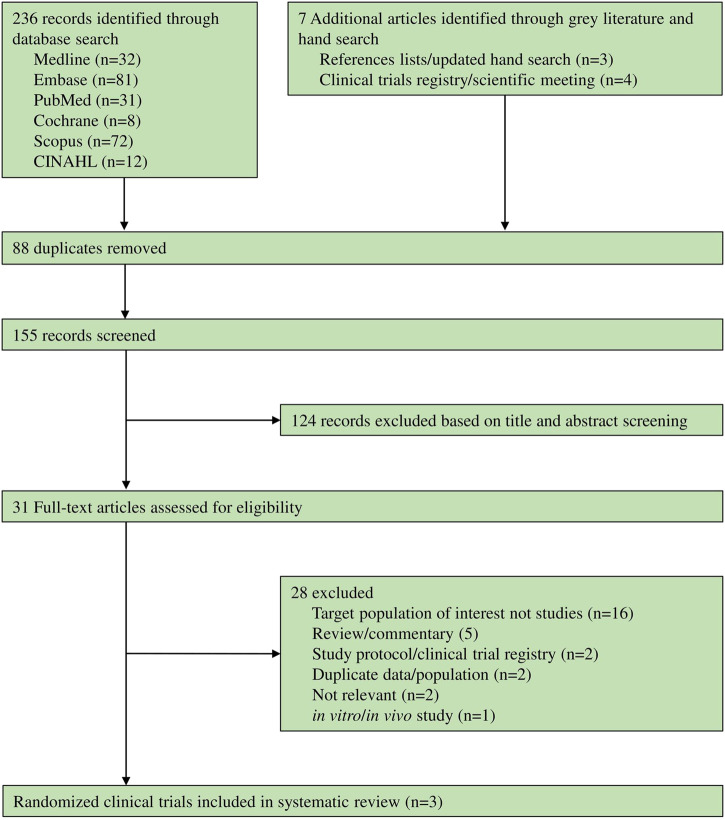
Flowchart of study selection.

236 records were identified through databases and seven articles were identified through grey literature and manual searches. Of these, 28 records were excluded (Supplementary Table S4), while only three RCTs (Ali Mapar and Namdari, 2019; Banavase Channakeshavaiah and Andanooru Chandrappa, 2020; AboAlsoud et al., 2022) met the eligibility criteria (shown in Figure 1).

Three included studies were prospective, monocentric, parallel-group RCTs conducted in Iran (*n* = 60) (Ali Mapar and Namdari, 2019), India (*n* = 40) (Banavase Channakeshavaiah and Andanooru Chandrappa, 2020), and Egypt (*n* = 40) (AboAlsoud et al., 2022). One RCT compared the efficacy of 15% metformin cream with a placebo for 12 weeks (Ali Mapar and Namdari, 2019). Another two RCTs investigated the efficacy of 30% topical metformin compared to those of TCC for melasma, with a follow-up time of 8 weeks. However, only topical formulations of metformin have been investigated for melasma.

As an intervention, one study used 15% metformin cream made from 15% metformin powder applied twice daily (Ali Mapar and Namdari, 2019), one study (Banavase Channakeshavaiah and Andanooru Chandrappa, 2020) used 30% metformin lotion made from 30 g of metformin applied at night daily, while another (AboAlsoud et al., 2022) used metformin 30% cream made from crushed metformin 500 mg tablets and applied at night daily. One RCT (Ali Mapar and Namdari, 2019) used a placebo as a comparator, whereas the other two RCTs (Banavase Channakeshavaiah and Andanooru Chandrappa, 2020; AboAlsoud et al., 2022) used TCC, which is a nighttime application of hydroquinone 2%, tretinoin 0.025%, and fluocinolone acetonide 0.01% (Table 1). The risk of bias of the three RCTs was rated as high due to inadequate information about allocation concealment and the randomization process. There were some concerns about outcome measurement because both assessors and patients were aware of the intervention, which could have led to detection bias (Supplementary Table S5).

### 3.1 Efficacy of topical metformin for melasma

One RCT (Ali Mapar and Namdari, 2019) assessed the effect of 15% topical metformin versus placebo during a 12-week follow-up time. At 8 weeks, there was no statistically significant difference in MASI score between the two groups (*p* = 0.79). However, when MASI score changes from baseline were measured, the MASI score in the metformin group decreased significantly compared to that in the placebo after 8 weeks (MD, −0.56; 95% CI, −1.07 to −0.04; *p* = 0.034). After 12 weeks, the difference in the MASI score changes became more apparent between the metformin and placebo groups (MD, −0.73; 95% CI, −1.25 to −0.20; *p* = 0.007) (Table 2). Regarding patient satisfaction, the mean satisfaction in the metformin group (3.66 ± 1.88) was significantly higher than that of the placebo group (1.06 ± 1.20, *p* < 0.001).

**TABLE 2:** Summary of findings and strength of evidence.

Melasma outcomes	No. of included trials (sample size)	Effect estimates (95% CI)	*p*-value	Heterogeneity				Strength of evidence (outcome classification)
				*Q* statistic	*p*-value	*I* ^*2*^ index (95% CI)	τ^2^	
(A) Compared with active-controlled (triple combination cream: Kligman’s formula)								
Treatment efficacy at 8 weeks								
Change in the MASI score from baseline	2 (80)	MD: 0.19 (−0.25–0.63)	0.390	0.58	0.448	0.0% (NA)	<0.001	Low (trivial, not different from Kligman’s formula)
Moderate to total global improvement (improvement in MASI score >25%)	2 (80)	OR: 0.90 (0.30–2.72)	0.848	1.08	0.299	7.3% (NA)	0.073	Low (trivial, not different from Kligman’s formula)
Treatment satisfaction: satisfied to highly satisfied	2 (80)	OR: 1.00 (0.23–4.31)	1.000	<0.01	1.000	0.0% (NA)	<0.001	Low (trivial, not different from Kligman’s formula)
Safety profiles at 8 weeks								
Unacceptability of treatment (all-cause study dropout)	2 (80)	Not estimated (no participant dropout during trial follow-up of 8 weeks)						Insufficient data
Tolerability (dropout due to adverse events)	2 (80)	Not estimated (no participant dropout during trial follow-up of 8 weeks)						Insufficient data
Serious adverse events	2 (80)	Not estimated (no participant dropout during trial follow-up of 8 weeks)						Insufficient data
Any adverse events	2 (80)	OR: 0.32 (0.07–1.51)	0.148	0.51	0.474	0.0% (NA)	<0.001	Low (trivial, not different from Kligman’s formula)
(B) Compared with placebo								
Treatment efficacy								
Change in the MASI score from baseline at 8 weeks	1 (60)	MD: −0.56 (−1.07 to −0.04)	0.034	NA	NA	NA	NA	Very low (beneficial with topical metformin)
Change in the MASI score from baseline at 12 weeks	1 (60)	MD: −0.73 (−1.25 to −0.20)	0.007	NA	NA	NA	NA	Very low (beneficial with topical metformin)
Safety profiles at 12 weeks								
Unacceptability of treatment (all-cause study dropout)	1 (60)	Not estimated (no participant dropout during trial follow-up of 12 weeks)						Insufficient data
Tolerability (dropout due to adverse events)	1 (60)	Not estimated (no participant dropout during trial follow-up of 12 weeks)						Insufficient data
Serious adverse events	1 (60)	Not estimated (no specific serious adverse events reported)						Insufficient data
Any adverse events	1 (60)	Not estimated (no specific adverse events reported)						Insufficient data

Abbreviations: CI, confidence interval; MASI, melasma area and severity index; NA, not applicable; OR, odds ratio; MD, mean difference.

**FIGURE 2:**
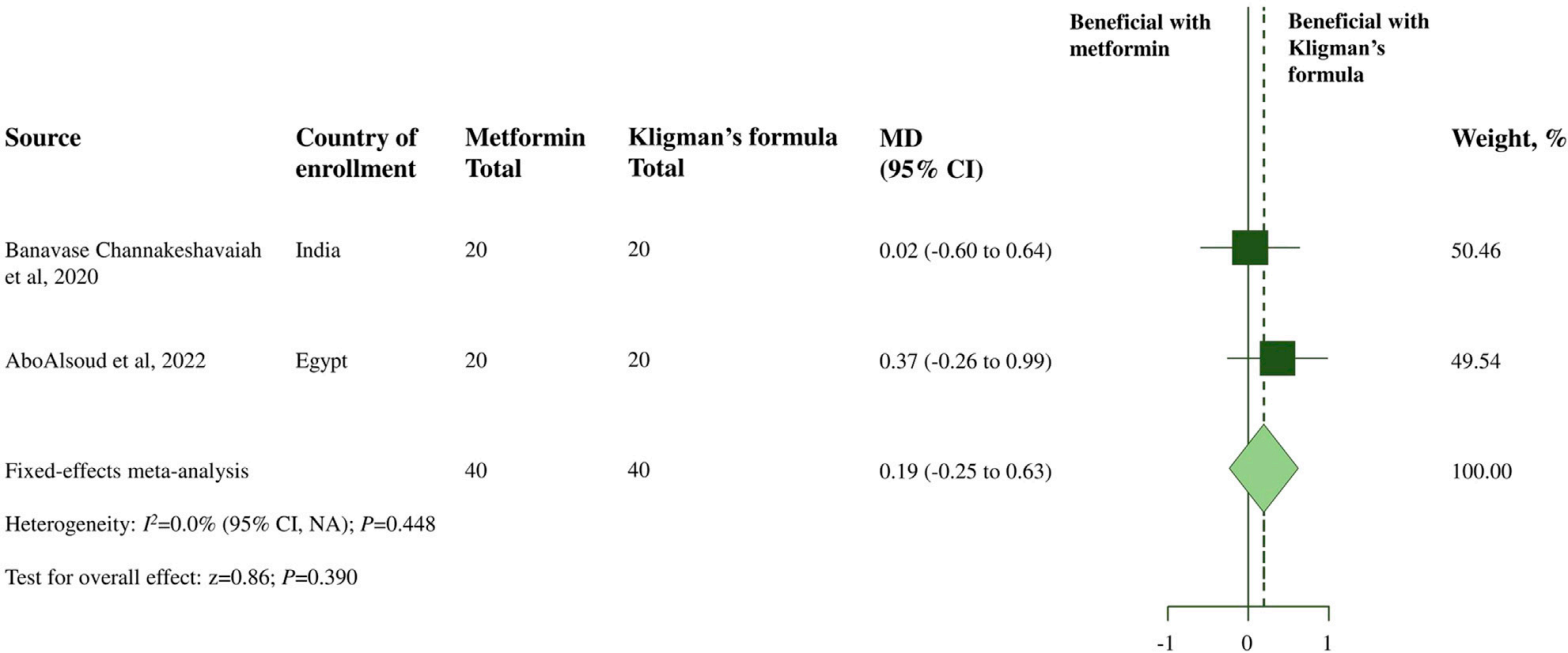
Meta-analysis of treatment efficacy of topical metformin vs. Topical combination cream (Kligman’s formula): change in the MASI score from baseline.

Moreover, two RCTs (Banavase Channakeshavaiah and Andanooru Chandrappa, 2020; AboAlsoud et al., 2022) assessed the severity and improvement of melasma in the topical metformin and TCC groups using the MASI score changes before and after 8 weeks of treatment. A meta-analysis of the two RCTs revealed no significant difference in decreasing the MASI score after 8 weeks of treatment between 30% topical metformin and TCC (MD, 0.19; 95% CI, −0.25 to 0.63; *p* = 0.390; I^2^ = 0.0%; shown in Table 2; Figure 2). According to the global improvement scale, there was no statistically significant difference in moderate to total global improvement (improvement in MASI score >25%) between the topical metformin and TCC groups (pooled OR, 0.90; 95% CI, 0.30 to 2.72; *p* = 0.848; I^2^ = 7.3%). Furthermore, differences in treatment satisfaction from satisfied to highly satisfied were not statistically significant between the two treatment groups (pooled OR, 1.00; 95% CI; 0.23 to 4.31; *p* = 1.000; I^2^ = 0.0%).

### 3.2 Safety of topical metformin for melasma

**FIGURE 3:**
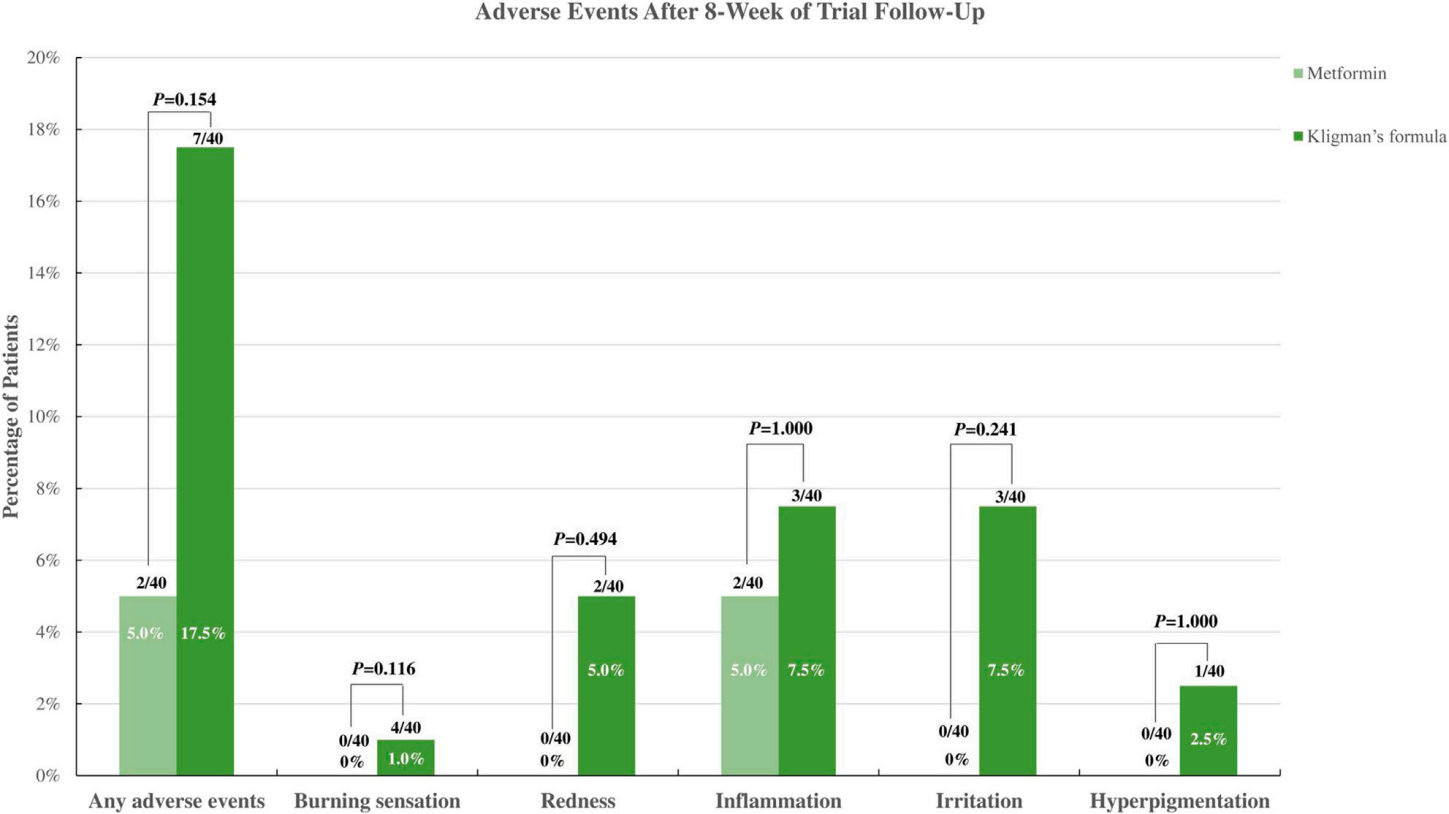
Incidence of adverse events of topical metformin vs. Topical combination cream (Kligman’s formula).

Regarding safety outcomes, according to a trial by Banavase Channakeshavaiah and Andanooru Chandrappa, 2020), there were no adverse effects observed in the 30% topical metformin group, whereas two patients in the TCC group complained of burning sensations, and one experienced both burning sensations and redness. A trial conducted by AboAlsoud et al., 2022 reported that only two patients in the topical metformin group experienced inflammation, while three patients experienced inflammation and irritation and one with redness, burning, and hyperpigmentation after using the TCC. When the incidence of adverse events was compared between the 30% topical metformin and TCC, patients applying metformin were less likely to experience adverse events. However, no statistically significant difference was observed (Figure 3). The meta-analysis also found no statistically significant difference in the occurrence of any adverse event between the 30% topical metformin and TCC groups (pooled OR, 0.32; 95% CI, 0.07 to 1.51; *p* = 0.148; I^2^ = 0.0%; Table 2).

### 3.3 Certainty of evidence assessment

The GRADE assessment for efficacy outcomes for topical metformin compared with placebo and topical metformin compared with TCC was rated as very low and low quality of evidence, respectively (Table 2). Furthermore, we graded the certainty of evidence regarding safety outcomes, as insufficient because of the limited data (Table 2; Supplementary Table S6).

## 4 Discussion and conclusion

This study evaluated the efficacy and safety of metformin for melasma. The results indicated that 15% topical metformin significantly decreased the MASI score from baseline compared with placebo after 8 weeks. Furthermore, when compared with TCC, topical metformin was as effective as TCC, although the certainty of the evidence was low.

Among the topical agents for melasma, TCC is considered the gold standard due to its potent whitening effect. Its efficacy is related to the synergistic effect of individual components. Tretinoin prevents hydroquinone oxidation and enhances epidermal penetration of other agents, whereas topical corticosteroids reduce cellular metabolism, inhibit melanin synthesis, and minimize irritation caused by the other two components (Kligman and Willis, 1975). However, erythema, burning, and irritation are common side effects (Mahajan et al., 2022), and such adverse events were found in this systematic review. Metformin has recently been used topically in hyperpigmentary disorders and perhaps in melasma owing to its molecular mechanism. Metformin reduces cAMP levels, which suppresses protein kinase A activity, leading to downregulation of the expression of the master gene for melanocyte survival (microphthalmia-associated transcription factor-MITF). Therefore, the transcription of melanogenic proteins such as tyrosinase, TRP-1, TRP-2, MART-1, and protein kinase C-beta (PKC-β) is reduced. Furthermore, metformin directly inhibits diacylglycerol and prevents PKC-β anchorage to melanosomes, thereby inhibiting melanogenesis (Park et al., 2004; Batchuluun et al., 2014; Belisle and Park, 2014; Bubna, 2016). Owing to these properties, metformin might be a potential medication to treat hyperpigmentation.

Based on our included studies, topical application was the only route of metformin administration available in clinical trials for melasma. The RCT comparing metformin with placebo used 15% topical metformin, whereas trials comparing topical metformin with TCC used 30% metformin. The utilization of 30% metformin might be supported by a previous *in-vitro* and *in-vivo* study in which 30% topical metformin was applied to mice’s tails and observed the depigmentation. The findings indicated that the topical metformin induced tail whitening in mice. They also confirmed the anti-melanogenic effect of metformin on the reconstituted human epidermis and human skin biopsies. They suggested a clinical strategy for using metformin for hyperpigmentation disorders (Lehraiki et al., 2014). In addition, patient satisfaction in the metformin group was significantly higher than that in the placebo or TCC group. This may be because of the few adverse events associated with metformin. A placebo-controlled trial also indicated that 15% topical metformin did not affect patients’ laboratory markers, such as fasting blood sugar, lipid profiles, and glomerular filtration rate after 12 weeks (Ali Mapar and Namdari, 2019). However, caution should be taken concerning the chronic use of metformin because long-term safety has not been studied.

Based on a previous meta-analysis comparing the efficacy of 14 melasma treatments, TCC was found to be the most favorable among the topical drugs for melasma (Liu et al., 2021). Moreover, a meta-analysis investigating the efficacy of topical agents for melasma by measuring the changes in pre- and post-treatment MASI scores concluded that non-hydroquinone agents may be considered as alternatives to hydroquinone-containing agents (Chang et al., 2023). However, neither study reported metformin as a melasma treatment. In addition, a previous review gathered studies on the use of metformin for dermatological diseases and concluded that oral metformin was effective and safe. It can also be considered an adjunctive treatment for psoriasis, hidradenitis suppurativa, polycystic ovarian syndrome-related acne, acanthosis, and hirsutism. However, metformin has not been mentioned for melasma treatment, and only oral metformin has been studied (Sung et al., 2020). In this circumstance, the use of metformin for melasma is a novel indication with limited clinical research. Therefore, the findings from this study can fill the knowledge gap in previous literature and provide a starting point for further investigation.

This study had limitations. First, despite the inclusion of RCTs, the bias was high. A high risk of bias was identified in the domain of the randomization process, and some concerns were raised regarding the outcome measurement. The authors did not clearly describe the randomization process or how the allocation sequence was concealed from investigators and patients. Furthermore, neither the patients nor the investigators in the two included RCTs that compared the efficacy of metformin and TCC were blinded. Knowledge of group regarding the assignment may influence their behavior in the trials and could lead to exaggeration of effect estimates. Second, a relatively small number of RCTs (*n* = 3 trials with a total of 140 participants) were included; the small study effect might have influenced our findings. Third, the results were based on a small sample size (40–60 patients). Finally, a few studies have reported the phenotypes, melasma type, severity, sun exposure, and stress, although these factors were cited as important factors affecting melasma (Espósito et al., 2022). We suggest that further studies should include the following minimum characteristics: (i) apply well-designed clinical trials with a clearly defined randomization process; (ii) include a large sample size with longer follow-up to establish the efficacy and safety of metformin for melasma, (iii) provide more information about the phenotypes, melasma type, severity, and risk factors for melasma, including current medication use; and (iv) evaluate the pigmentary alterations histologically and immunohistochemically. In addition, further research is needed to understand the specific mechanism of metformin in melasma and its efficacy in resistant or refractory melasma.

Our study had several strengths. This is the first systematic review and meta-analysis to investigate the efficacy and safety of metformin for melasma. The results of the current study shed further light on the understanding and potential use of metformin in treating melasma or depigmentation. In addition, we undertook a comprehensive search to ascertain that all relevant studies were included. Finally, our study adhered to the standard systematic review and meta-analysis methodology and reporting recommended by the Cochrane and PRISMA checklist statements (Page et al., 2021; Higgins JPT et al., 2022).

According to our findings, a 30% topical metformin may be a possible treatment option for melasma. This demonstrated depigmentation by reducing cAMP accumulation, which reduced the expression of MITF and other melanogenic proteins. It had fewer unfavorable adverse events and was more tolerable than TCC. However, current evidence relies on animal studies and a few clinical trials with small sample sizes. Currently, TCC is the most effective topical preparation for melasma. Evidence has revealed that TCC has been shown to improve or clear up to 60%–80% of patients with melasma (Doolan and Gupta, 2021). Therefore, TCC can be recommended as a first-line treatment; however, if patients have adverse events or if their melasma does not improve, new agents with the potential to inhibit melanogenesis, such as topical metformin, can be used in sequential therapy. It is also crucial to educate patients about melasma triggers, and the significance of daily sunscreen usage and maintenance treatment, to reduce the chance of recurrence (Shankar et al., 2014; Ogbechie-Godec and Elbuluk, 2017).

In summary, the current study found that topical metformin significantly reduced the MASI score compared with placebo. Compared to TCC, topical metformin was equally effective in decreasing changes in the MASI score in patients with melasma following an 8-week period with minimum adverse events and high satisfaction. However, a large sample size, longer follow-up time, and well-designed trials are required to confirm the efficacy and safety of metformin for melasma.

## Data Availability

The raw data supporting the conclusion of this article will be made available by the authors, without undue reservation.
