# Supplementary material for: Efficacy and safety of metformin for melasma treatment: a systematic review and meta-analysis
Source: Front Pharmacol. 2023 Dec 13;14:1281050. doi: 10.3389/fphar.2023.1281050 (PMC10773746; doi:10.3389/fphar.2023.1281050)
Supplement: Supplementary file 1 [file Table1.pdf]

Supplementary Material

Efficacy and Safety of Metformin for Melasma: A Systematic Review and Meta-Analysis

Pajaree Mongkhon, Chidchanok Ruengorn, Ratanaporn Awiphan, Chabaphai Phosuya, Yongyuth Ruanta, Kednapa Thavorn, Sirinda Jamjanya, Mati Chuamanochan*, Surapon Nochaiwong*

* Correspondence:

Surapon Nochaiwong

Department of Pharmaceutical Care, Faculty of Pharmacy, Chiang Mai University, Chiang Mai 50200, Thailand, Phone: +66-899973365, Fax: 6653222741

Email: surapon.nochaiwong@gmail.com

or

Mati Chuamanochan

Division of Dermatology, Department of Internal Medicine, Faculty of Medicine, Chiang Mai University, Chiang Mai 50200, Thailand, Phone: +66-53936735-6, Fax: +66-53946234

Email: mati.c@cmu.ac.th

Supplementary Online Content

**Table S1**	Systematic Review Search Strategy	S2
**Table S2**	Grey Literature Search	S8
**Table S3**	PICOTS: Inclusion and Exclusion Criteria	S9
**Table S4**	Excluded Studies	S10
**Table S5**	Summary Results of Risk-of-Bias Assessment	S12
**Table S6**	Quality of Evidence Synthesis and GRADE Evidence Profile of Outcomes	S13
**Appendix I**	PRISMA 2020 Statement Checklist	S16

Table S1 Systematic Review Search Strategy

**Medline via OVID (From Inception to October 4, 2022)**		
**Search**	**Query**	**Items Found**
#1	exp melasma/	8958
#2	melasma.mp.	1522
#3	exp melanosis/	8958
#4	melanosis.mp.	4999
#5	exp chloasma/	8958
#6	chloasma.mp.	183
#7	(melasma or melanosis or chloasma or hyperpigment*).tw,kw,rn.	13213
#8	or/1-7	19372
#9	exp metformin/	16905
#10	metformin.mp.	27842
#11	exp biguanides/	31442
#12	(biguanid* or dimethylguanylguanidine or dimethylbiguanidine).mp.	5580
#13	(Glucophag* or Riomet or Fortamet or Glumetza or Obimet or Dianben or Diabex or Diaformin or Diabetosan or Fluamine or Flumamine or Glifage or Gliguanid or Haurymelin or Imidodicarbonimidic diamide or Islotin or LA-6023 or CCRIS 9321 or DMGG or EINECS 211-517-8 or Melbin or Metiguanide or Siofor or UNII-9100L32L2N).tw,kw,rn.	166
#14	or/9-13	43267
#15	8 and 14	50
#16	(news or newspaper article or letter or interview comment or editorial or review or systematic review or meta-analysis).pt.	5222331
#17	15 not 16	35
#18	limit 17 to human	32

Table S1 Systematic Review Search Strategy (Continued)

**Embase via OVID (From Inception to October 4, 2022)**		
**Search**	**Query**	**Items Found**
#1	exp melasma/	2901
#2	melasma.mp.	2439
#3	exp melanosis/	3903
#4	melanosis.mp.	4826
#5	exp chloasma/	2901
#6	chloasma.mp.	2962
#7	(melasma or melanosis or chloasma or hyperpigment*).tw,kw,rn.	19328
#8	or/1-7	21790
#9	exp metformin/	78424
#10	metformin.mp.	82142
#11	exp biguanides/	115277
#12	(biguanid* or dimethylguanylguanidine or dimethylbiguanidine).mp.	9051
#13	(Glucophag* or Riomet or Fortamet or Glumetza or Obimet or Dianben or Diabex or Diaformin or Diabetosan or Fluamine or Flumamine or Glifage or Gliguanid or Haurymelin or Imidodicarbonimidic diamide or Islotin or LA-6023 or CCRIS 9321 or DMGG or EINECS 211-517-8 or Melbin or Metiguanide or Siofor or UNII-9100L32L2N).tw,kw,rn.	2033
#14	or/9-13	118662
#15	8 and 14	111
#16	(news or newspaper article or letter or interview comment or editorial or review or systematic review or meta-analysis).pt.	4936821
#17	15 not 16	89
#18	limit 17 to human	81

Table S1 Systematic Review Search Strategy (Continued)

**PubMed (From Inception to October 4, 2022)**		
**Search**	**Query**	**Items Found**
#1	melasma[MeSH Terms]	8956
#2	melanosis[MeSH Terms]	8956
#3	chloasma[MeSH Terms]	8956
#4	melasma[Title/Abstract] OR melanosis[Title/Abstract] OR chloasma[Title/Abstract] OR hyperpigment*[Title/Abstract]	13446
#5	#1 OR #2 OR #3 OR #4	19536
#6	metformin[MeSH Terms]	16867
#7	biguanides[MeSH Terms]	31397
#8	metformin*[Title/Abstract] OR biguanid*[Title/Abstract] OR dimethylguanylguanidine[Title/Abstract] OR dimethylbiguanidine[Title/Abstract]	28261
#9	Glucophag*[Title/Abstract] OR Riomet[Title/Abstract] OR Fortamet[Title/Abstract] OR Glumetza[Title/Abstract] OR Obimet[Title/Abstract] OR Dianben[Title/Abstract] OR Diabex[Title/Abstract] OR Diaformin[Title/Abstract] OR Diabetosan[Title/Abstract] OR Fluamine[Title/Abstract] OR Flumamine[Title/Abstract] OR Glifage[Title/Abstract] OR Gliguanid[Title/Abstract] OR Haurymelin[Title/Abstract] OR "Imidodicarbonimidic diamide"[Title/Abstract] OR Islotin[Title/Abstract] OR "LA-6023"[Title/Abstract] OR "CCRIS 9321"[Title/Abstract] OR DMGG[Title/Abstract] OR "EINECS 211-517-8"[Title/Abstract] OR Melbin[Title/Abstract] OR Metiguanide[Title/Abstract] OR Siofor[Title/Abstract] OR "UNII-9100L32L2N"[Title/Abstract]	167
#10	#6 OR #7 OR #8 OR #9	43238
#11	#5 AND #10	50
#12	Comment[Publication Type] OR Editorial[Publication Type] OR Guideline[Publication Type] OR Letter[Publication Type] OR News[Publication Type] OR Newspaper Article[Publication Type] OR Review[Publication Type] OR "Systematic Review"[Publication Type] OR "Meta-Analysis"[Publication Type]	5502836
#13	#11 NOT #12	34
#14	Filters applied: Humans	31

Table S1 Systematic Review Search Strategy (Continued)

**Cochrane Library (From Inception to October 4, 2022)**		
**Search**	**Query**	**Items Found**
#1	MeSH descriptor: [Melanosis] explode all trees	372
#2	(melasma OR melanosis OR chloasma OR hyperpigment*):ti,ab,kw	2091
#3	#1 OR #2	2132
#4	MeSH descriptor: [Metformin] explode all trees	4543
#5	MeSH descriptor: [Biguanides] explode all trees	7311
#6	(metformin* OR biguanid* OR dimethylguanylguanidine OR dimethylbiguanidine):ti,ab,kw	12604
#7	(Glucophag* OR Riomet OR Fortamet OR Glumetza OR Obimet OR Dianben OR Diabex OR Diaformin OR Diabetosan OR Fluamine OR Flumamine OR Glifage OR Gliguanid OR Haurymelin OR "Imidodicarbonimidic diamide" OR Islotin OR "LA 6023" OR "CCRIS 9321" OR DMGG OR "EINECS 2115178" OR Melbin OR Metiguanide OR Siofor OR "UNII 9100L32L2N"):ti,ab,kw	267
#8	#4 OR #5 OR #6 OR #7	15193
#9	#3 AND #8	8
#10	Limit in Trials	8

Table S1 Systematic Review Search Strategy (Continued)

**Scopus (From Inception to October 4, 2022)**		
**Search**	**Query**	**Items Found**
#1	TITLE-ABS-KEY (melasma)	2083
#2	TITLE-ABS-KEY (melanosis)	6715
#3	TITLE-ABS-KEY (chloasma)	2351
#4	TITLE-ABS-KEY (hyperpigment*)	25927
#5	#1 OR #2 OR #3 OR #4	32664
#6	TITLE-ABS-KEY (metformin* OR biguanide* OR dimethylguanylguanidine OR dimethylbiguanidine)	75659
#7	TITLE-ABS-KEY (glucophag* OR riomet OR fortamet OR glumetza OR obimet OR dianben OR diabex OR diaformin OR diabetosan OR fluamine OR flumamine OR glifage OR gliguanid OR haurymelin OR "Imidodicarbonimidic diamide" OR islotin OR "LA-6023" OR "CCRIS 9321" OR dmgg OR "EINECS 211-517-8" OR melbin OR metiguanide OR siofor OR "UNII-9100L32L2N")	2007
#8	#6 OR #7	75708
#9	#5 AND #8	109
#10	Filters: (EXCLUDE (DOCTYPE , "re") OR EXCLUDE (DOCTYPE , "le") OR EXCLUDE (DOCTYPE , "no") OR EXCLUDE (DOCTYPE , "cp") OR EXCLUDE (DOCTYPE , "sh")) AND (EXCLUDE (SRCTYPE , "k"))	72

Table S1 Systematic Review Search Strategy (Continued)

**CINAHL (From Inception to October 4, 2022)**		
**Search**	**Query**	**Items Found**
#1	MW melanosis	924
#2	AB melasma OR melanosis OR chloasma OR hyperpigment*	1564
#3	S1 OR S2	2235
#4	MW metformin	6439
#5	AB metformin* OR biguanid* OR dimethylguanylguanidine OR dimethylbiguanidine	6054
#6	AB Glucophag* OR Riomet OR Fortamet OR Glumetza OR Obimet OR Dianben OR Diabex OR Diaformin OR Diabetosan OR Fluamine OR Flumamine OR Glifage OR Gliguanid OR Haurymelin OR "Imidodicarbonimidic diamide" OR Islotin OR "LA 6023" OR "CCRIS 9321" OR DMGG OR "EINECS 2115178" OR Melbin OR Metiguanide OR Siofor OR "UNII 9100L32L2N"	30
#7	S4 OR S5 OR S6	9041
#8	S3 AND S7	13
#9	MW animal OR in vivo OR in vitro	185747
#10	S8 NOT S9	13
#11	Expanders - Apply equivalent subjects<br>Source Types - Academic Journals	12

Table S2 Grey Literature Search

Ongoing clinical trial register

- Australia and New Zealand's (ANZCTR) (http://www.anzctr.org.au)
- Brazilian Clinical Trials Registry (ReBec) (http://www.ensaiosclinicos.gov.br)
- Chinese Clinical Trial Registry (ChiCTR) (http://www.chictr.org.cn)
- Clinical Research Information Service (CRiS), Republic of Korea (http://cris.cdc.go.kr)
- Clinical Trials Registry - India (CTRI) (http://ctri.nic.in)
- Cuban Public Registry of Clinical Trials(RPCEC) (http://registroclinico.sld.cu)
- EU Clinical Trials Register (EU-CTR) (https://www.clinicaltrialsregister.eu)
- German Clinical Trials Register (DRKS) (http://www.drks.de)
- Iranian Registry of Clinical Trials (IRCT) (http://www.irct.ir)
- Japan Primary Registries Network (https://rctportal.niph.go.jp)
- The Netherlands Trial Register (http://www.trialregister.nl)
- Pan African Clinical Trial Registry (PACTR) (http://www.pactr.org)
- Peruvian Registry of Clinical Trials (http://www.ins.gob.pe/ensayosclinicos)
- Philippine Health Research Registry (http://registry.healthresearch.ph)
- Sri Lanka Clinical Trials Registry (SLCTR) (http://www.slctr.lk)
- South African National Clinical Trials Register (http://www.sanctr.gov.za)
- Swiss FOPH Human Research Projects (https://www.kofam.ch/en/swiss-clinical-trials-portal.html)
- Tanzania Clinical Trial Registry (http://www.tzctr.or.tz)
- Thai Clinical Trials Registry (http://www.clinicaltrials.in.th)
- The United Kingdoms' ISRCTN registry (http://www.isrctn.com)
- The US National Institutes of Health Ongoing Trials Registry (http://clinicaltrials.gov)
- The World Health Organization International Clinical Trials Registry Platform (ICTRP) (https://www.who.int/ictrp)

Preprint databases

- medRxiv (https://www.medrxiv.org)
- bioRxiv (https://www.biorxiv.org)
- Research Square (https://www.researchsquare.com)

Table S3 Study Inclusion and Exclusion Criteria

Category	Criteria for inclusion	Criteria for exclusion
Populations	• Adults participants aged 18 years or older diagnosed with melasma regardless of sex, race, and comorbidities	• Studies that recruiting participants aged less than 18 years<br>• Studies including participants with post-inflammatory hyperpigmentation, neurodermatitis, eczema, atrophy, rosacea, or pregnant/lactating women
Interventions	• Metformin in any route of administration	• Treatment comparisons other than metformin therapy
Comparators	• Placebo/vehicle, active comparator, different dosage regimen, or usual care	• Studies without control groups
Outcomes	• Treatment efficacy<br>○ Change in MASI score from baseline<br>○ Global improvement<br>○ Treatment satisfaction<br>• Safety outcomes<br>○ Unacceptability of treatment (study discontinuation due to any cause)<br>○ Tolerability (study discontinuation due to adverse events)<br>○ Any adverse events<br>○ Adverse skin reactions	• Studies not providing data for calculate the primary or secondary outcomes<br>• Studies with a follow-up period of less than one week
Timing	• An extensive search strategy from the inception of bibliographic databases forward to assure all published literature was identified	• No limit timing of start date<br>• No language restrictions
Setting	• Published RCTs, quasi-RCTs, observational studies (cohort, case-control, cross-sectional), case series, and case report in any setting and context	• *in vitro*/*in vivo* or animal studies<br>• Review and systematic review

Abbreviations: MASI; Melasma Area and Severity Index; RCTs, randomized controlled trials.

Table S4 Excluded Studies

Author, Year	Title	Reason for Exclusion
Hermanns-Lê et al, 2002	Juvenile acanthosis nigricans and insulin resistance. Pediatr Dermatol. 2002 Jan-Feb;19(1):12-4.	Target population of interest not studies
Tankova et al, 2002	Therapeutic approach in insulin resistance with acanthosis nigricans. Int J Clin Pract. 2002 Oct;56(8):578-81.	Target population of interest not studies
Walling et al, 2003	Improvement of acanthosis nigricans on isotretinoin and metformin. J Drugs Dermatol. 2003 Dec;2(6):677-81.	Target population of interest not studies
Hermanns-Lê et al, 2004	Acanthosis nigricans associated with insulin resistance: pathophysiology and management. Am J Clin Dermatol. 2004;5(3):199-203.	Review article
Bellot-Rojas et al, 2006	Comparison of metformin versus rosiglitazone in patients with Acanthosis nigricans: a pilot study. J Drugs Dermatol. 2006 Oct;5(9):884-9.	Target population of interest not studies
Inagaki et al, 2006	Metformine hydrochloride reduces both acanthosis nigricans and insulin resistance in Japanese young female. Nihon Naika Gakkai Zasshi. 2006 Dec 10;95(12):2550-2.	Target population of interest not studies
Banecka et al, 2007	Metabolic syndrome in a 15-year-old girl with PCOS and acanthosis nigricans...polycystic ovary syndrome. Experimental & Clinical Diabetology / Diabetologia Doswiadczalna i Kliniczna. 2007;7(5):256-259.	Target population of interest not studies
Valizadeh et al, 2008	Severe acanthosis nigricans in a 17 year-old female with partial lipodystrophic syndrome. J Pediatr Endocrinol Metab. 2008 Nov;21(11):1027-8.	Target population of interest not studies
Vinzio et al, 2009	Acanthosis nigricans. Medecine des Maladies Metaboliques 2009; 3: 519.	Commentary/short review
Click et al, 2011	Facial Acanthosis Nigricans. Consultant (00107069). 2011;51(12):937.	Target population of interest not studies
Balaji et al, 2014	Significance of acanthosis nigricans as marker for metabolic syndrome. World Journal of Medical Sciences 2014;10(3):295-8.	Target population of interest not studies
Belisle et al, 2014	Metformin: a potential drug to treat hyperpigmentation disorders. J Invest Dermatol. 2014 Oct;134(10):2488-2491.	Commentary
Lehraiki et al, 2014	Inhibition of melanogenesis by the antidiabetic metformin. J Invest Dermatol. 2014 Oct;134(10):2589-2597.	in vitro/in vivo study
ClinicalTrials.gov, 2015	Study of Efficacy of Metformin in the Treatment of Acanthosis Nigricans in Children With Obesity. https://clinicaltrials.gov/show/NCT02438020.	Study protocol/clinical trial registry
Bubna, 2016	Metformin - For the dermatologist. Indian J Pharmacol. 2016 Jan-Feb;48(1):4-10.	Review article
Kępczyńska-Nyk et al, 2016	Woman 19-old with hirsutism, obesity and acanthosis nigricans. Pol Merkur Lekarski. 2016 Sep 29;41(243):141-144.	Target population of interest not studies

Table S4 Excluded Studies (Continued)

Author, Year	Title	Reason for Exclusion
Giri et al, 2017	Acanthosis Nigricans and Its Response to Metformin. Pediatr Dermatol. 2017 Sep;34(5):e281-e282.	Target population of interest not studies
Mahjour et al, 2017	The role of oligomenorrhea in melasma. Med Hypotheses. 2017 Jul;104:1-3.	Not relevant
Singh et al, 2017	Randomized Placebo Control Study of Metformin in Psoriasis Patients with Metabolic Syndrome (Systemic Treatment Cohort). Indian J Endocrinol Metab. 2017 Jul-Aug;21(4):581-587.	Target population of interest not studies
International Clinical Trials Registry Platform, 2018	Treatment for melasma by metformin lotion. https://trialsearch.who.int/Trial2.aspx?TrialID=CTRI/2018/12/016588.	Duplicate population with Banavase Channakeshavaiah, et al (2020)
ClinicalTrials.gov, 2018	Use the systemic metformin in melasma. https://clinicaltrials.gov/show/NCT03475524.	Study protocol/clinical trial registry
Dumaguin et al, 2019	Metformin-induced photocontact dermatitis in a 67-year-old male: A case report. Phillippine Journal of Internal Medicine 2019; 57: 103-6.	Not relevant
Ozdemir Kutbay et al, 2019	An unusual case of acquired partial lipodystrophy presenting with acanthosis nigricans. Acta Endocrinol (Buchar). 2019 Jan-Mar;-5(1):129-130.	Target population of interest not studies
Sett et al, 2019	Effectiveness and Safety of Metformin versus Canthex™ in Patients with Acanthosis Nigricans: A Randomized, Double-blind Controlled Trial. Indian J Dermatol. 2019 Mar-Apr;64(2):115-121.	Target population of interest not studies
Langar et al, 2020	To evaluate the safety and efficacy of oral isotretinoin with jessner's-35%trichloroacetic acid peel (Monheit Technique) for the treatment of acanthosis nigricans in coloured skin. J Dermatol Nurses Assoc. Conference: 24th World Congress of Dermatology. Milan, Italy	Duplicate population with Langar (2020)
Langar, 2021	Safety and efficacy of Jessner's- 35%Trichloroacetic acid peel (Monheit's Technique) with oral isotretinoin in the treatment of extra facial acanthosis nigricans in skin of color. Pigment Cell Melanoma Res. 2021;34(2):472.	Target population of interest not studies
Sinha, 2021	Evaluation of TCA peels in non-malignant acanthosis nigricans. Pigment Cell Melanoma Res. 2021;34(2):472.	Target population of interest not studies
Monte-Serrano et al, 2022	The role of metformin in the treatment of dermatological diseases: A narrative review. Aten Primaria. 2022 Jun;54(6):102354.	Review article

Table S5 Summary Results of Risk-of-Bias Assessment

**Bias Domain: Version 2 of the Cochrane Risk-of-Bias Assessment Tool for Randomized Trials**	**Author, Year**		
	**Mapar *et al* (2019)**	**Banavase Channakeshavaiah *et al*, 2020**	**AboAlsoud *et al*, 2022**
Randomization process	High risk	High risk	High risk
Deviations from intended interventions	Some concerns	Low risk	Low risk
Missing outcome data	Some concerns	Low risk	Low risk
Measurement of the outcome	Some concerns	Some concerns	Some concerns
Selection of the reported result	High risk	Some concerns	Some concerns
Overall risk-of-bias	High	High	High
	-	-	-

Table S6 Quality of Evidence Synthesis and GRADE Evidence Profile of Outcomes

Outcomes	No. of Studies	Study Design (Sample Size)	Quality Assessment: Required Domains					Other Issues	Finding and Direction (Magnitude) of Effect	Strength of Evidence
			Study Limitations	Directions	Consistency	Precision	Reporting Bias			
**(A) Compared with active-controlled (triple combination cream: Kligman's formula)**										
Change in MASI score from baseline	2	RCTs (80)	High	Direct	Consistency	Imprecision	Undetected	Bias arising from the randomization process and there were some concerns about outcome measurement which could lead to detection bias.	Two RCTs with high study limitations. The precision is low as the effect estimates come from only two RCTs with small sample size. The results were consistent due to the overlapped CIs and low heterogeneity.	Low (trivial, not different from Kligman's formula)
Moderate to total global improvement (improvement in MASI score >25%)	2	RCTs (80)	High	Direct	Consistency	Imprecision	Undetected	Bias arising from the randomization process and there were some concerns about outcome measurement which could lead to detection bias.	Two RCTs with high study limitations. The precision is low as the effect estimates come from only two RCTs with small sample size. The results were consistent due to low heterogeneity.	Low (trivial, not different from Kligman's formula)
Treatment satisfaction: satisfied to highly satisfied	2	RCTs (80)	High	Direct	Consistency	Imprecision	Undetected	Bias arising from the randomization process and there were some concerns about outcome measurement which could lead to detection bias.	Two RCTs with high study limitations. The precision is low as the effect estimates come from only two RCTs with small sample size. The results were consistent due to low heterogeneity.	Low (trivial, not different from Kligman's formula)

Unacceptability of treatment (all-cause study dropout)	2	RCTs (80)	High	Direct	Unknown	Unknown	Unknown	Bias arising from the randomization process and outcome measurement.	No participant dropout during trial follow-up of 8 weeks.	Insufficient data
Tolerability (dropout due to adverse events)	2	RCTs (80)	High	Direct	Unknown	Unknown	Unknown	Bias arising from the randomization process and outcome measurement.	No participant dropout during trial follow-up of 8 weeks.	Insufficient data
Serious adverse events	2	RCTs (80)	High	Direct	Unknown	Unknown	Unknown	Bias arising from the randomization process and outcome measurement.	No participant dropout during trial follow-up of 8 weeks.	Insufficient data
Any adverse events	2	RCTs (80)	High	Direct	Consistency	Imprecision	Undetected	Bias arising from the randomization process and outcome measurement.	Two RCTs with high study limitations. The precision is low as the effect estimates come from only two RCTs with small sample size. The results were consistent due to low heterogeneity.	Low (trivial, not different from Kligman's formula)
**(B) Compared with placebo**										
Change in MASI score from baseline at 8 weeks	1	RCTs (60)	High	Direct	Inconsistency	Imprecision	Suspected	Bias arising from the randomization process, outcome measurement, and selection of the reported result would affect observed effect.	The results relied on only one RCT with a small sample size.	Very low (beneficial with topical metformin)
Change in MASI score from	1	RCTs (60)	High	Direct	Inconsistency	Imprecision	Suspected	Bias arising from the randomization process, outcome measurement, and	The results relied on only one RCT with a small sample size.	Very low (beneficial with topical metformin)

baseline at 12 weeks								selection of the reported result would affect observed effect.		
Unacceptability of treatment (all-cause study dropout)	1	RCTs (60)	High	Direct	Unknown	Unknown	Unknown	Bias arising from the randomization process, outcome measurement, and selection of the reported result would affect observed effect.	No participant dropout during trial follow-up of 12 weeks.	Insufficient data
Tolerability (dropout due to adverse events)	1	RCTs (60)	High	Direct	Unknown	Unknown	Unknown	Bias arising from the randomization process, outcome measurement, and selection of the reported result would affect observed effect.	No participant dropout during trial follow-up of 12 weeks.	Insufficient data
Serious adverse events	1	RCTs (60)	High	Direct	Unknown	Unknown	Unknown	Bias arising from the randomization process, outcome measurement, and selection of the reported result would affect observed effect.	No specific serious adverse events reported	Insufficient data
Any adverse events	1	RCTs (60)	High	Direct	Unknown	Unknown	Unknown	Bias arising from the randomization process, outcome measurement, and selection of the reported result would affect observed effect.	No specific adverse events reported.	Insufficient data

Abbreviations: RCTs, randomized controlled trials; CIs, confidence interval; GRADE, Grading of Recommendations Assessment, Development and Evaluation.

Appendix I PRISMA 2020 Statement Checklist

Section and Topic	Item #	Checklist item	Has the item been reported or not?
**TITLE**			
Title	1	Identify the report as a systematic review.	✓
**ABSTRACT**			
Abstract	2	See the PRISMA 2020 for Abstracts checklist.	✓
**INTRODUCTION**			
Rationale	3	Describe the rationale for the review in the context of existing knowledge.	✓
Objectives	4	Provide an explicit statement of the objective(s) or question(s) the review addresses.	✓
**METHODS**			
Eligibility criteria	5	Specify the inclusion and exclusion criteria for the review and how studies were grouped for the syntheses.	✓ Table S3
Information sources	6	Specify all databases, registers, websites, organisations, reference lists and other sources searched or consulted to identify studies. Specify the date when each source was last searched or consulted.	✓
Search strategy	7	Present the full search strategies for all databases, registers and websites, including any filters and limits used.	Table S1-S2
Selection process	8	Specify the methods used to decide whether a study met the inclusion criteria of the review, including how many reviewers screened each record and each report retrieved, whether they worked independently, and if applicable, details of automation tools used in the process.	✓
Data collection process	9	Specify the methods used to collect data from reports, including how many reviewers collected data from each report, whether they worked independently, any processes for obtaining or confirming data from study investigators, and if applicable, details of automation tools used in the process.	✓
Data items	10a	List and define all outcomes for which data were sought. Specify whether all results that were compatible with each outcome domain in each study were sought (e.g. for all measures, time points, analyses), and if not, the methods used to decide which results to collect.	✓
	10b	List and define all other variables for which data were sought (e.g. participant and intervention characteristics, funding sources). Describe any assumptions made about any missing or unclear information.	✓
Study risk of bias assessment	11	Specify the methods used to assess risk of bias in the included studies, including details of the tool(s) used, how many reviewers assessed each study and whether they worked independently, and if applicable, details of automation tools used in the process.	✓
Effect measures	12	Specify for each outcome the effect measure(s) (e.g. risk ratio, mean difference) used in the synthesis or presentation of results.	✓
Synthesis methods	13a	Describe the processes used to decide which studies were eligible for each synthesis (e.g. tabulating the study intervention characteristics and comparing against the planned groups for each synthesis (item #5)).	✓
	13b	Describe any methods required to prepare the data for presentation or synthesis, such as handling of missing summary statistics, or data conversions.	✓
	13c	Describe any methods used to tabulate or visually display results of individual studies and syntheses.	✓
	13d	Describe any methods used to synthesize results and provide a rationale for the choice(s). If meta-analysis was performed, describe the model(s), method(s) to identify the presence and extent of statistical heterogeneity, and software package(s) used.	✓

Section and Topic	Item #	Checklist item	Has the item been reported or not?
	13e	Describe any methods used to explore possible causes of heterogeneity among study results (e.g. subgroup analysis, meta-regression).	✓
	13f	Describe any sensitivity analyses conducted to assess robustness of the synthesized results.	-
Reporting bias assessment	14	Describe any methods used to assess risk of bias due to missing results in a synthesis (arising from reporting biases).	NA
Certainty assessment	15	Describe any methods used to assess certainty (or confidence) in the body of evidence for an outcome.	✓
**RESULTS**			
Study selection	16a	Describe the results of the search and selection process, from the number of records identified in the search to the number of studies included in the review, ideally using a flow diagram.	✓ Figure 1
	16b	Cite studies that might appear to meet the inclusion criteria, but which were excluded, and explain why they were excluded.	✓ Table S4
Study characteristics	17	Cite each included study and present its characteristics.	✓ Table 1
Risk of bias in studies	18	Present assessments of risk of bias for each included study.	✓ Table 1, Table S5
Results of individual studies	19	For all outcomes, present, for each study: (a) summary statistics for each group (where appropriate) and (b) an effect estimate and its precision (e.g. confidence/credible interval), ideally using structured tables or plots.	✓ Table 2, Figure 2
Results of syntheses	20a	For each synthesis, briefly summarise the characteristics and risk of bias among contributing studies.	✓
	20b	Present results of all statistical syntheses conducted. If meta-analysis was done, present for each the summary estimate and its precision (e.g. confidence/credible interval) and measures of statistical heterogeneity. If comparing groups, describe the direction of the effect.	✓ Table 2, Figure 2
	20c	Present results of all investigations of possible causes of heterogeneity among study results.	NA
	20d	Present results of all sensitivity analyses conducted to assess the robustness of the synthesized results.	NA
Reporting biases	21	Present assessments of risk of bias due to missing results (arising from reporting biases) for each synthesis assessed.	NA
Certainty of evidence	22	Present assessments of certainty (or confidence) in the body of evidence for each outcome assessed.	✓ Table S6
**DISCUSSION**			
Discussion	23a	Provide a general interpretation of the results in the context of other evidence.	✓
	23b	Discuss any limitations of the evidence included in the review.	✓
	23c	Discuss any limitations of the review processes used.	✓
	23d	Discuss implications of the results for practice, policy, and future research.	✓
**OTHER INFORMATION**			
Registration and protocol	24a	Provide registration information for the review, including register name and registration number, or state that the review was not registered.	✓

Section and Topic	Item #	Checklist item	Has the item been reported or not?
	24b	Indicate where the review protocol can be accessed, or state that a protocol was not prepared.	✓
	24c	Describe and explain any amendments to information provided at registration or in the protocol.	✓
Support	25	Describe sources of financial or non-financial support for the review, and the role of the funders or sponsors in the review.	✓
Competing interests	26	Declare any competing interests of review authors.	✓
Availability of data, code and other materials	27	Report which of the following are publicly available and where they can be found: template data collection forms; data extracted from included studies; data used for all analyses; analytic code; any other materials used in the review.	NA

Page MJ, McKenzie JE, Bossuyt PM, Boutron I, Hoffmann TC, Mulrow CD, et al. The PRISMA 2020 statement: an updated guideline for reporting systematic reviews. BMJ 2021;372:n71.
